# Measuring the potential of individual airports for pandemic spread over the world airline network

**DOI:** 10.1186/s12879-016-1350-4

**Published:** 2016-02-09

**Authors:** Glenn Lawyer

**Affiliations:** Department of Computational Biology, Max Planck Institute for Informatics, Campus E1 4, Saarbrücken, Germany

**Keywords:** Epidemic, Pandemic, Airline, Network, Stochastic, Centrality

## Abstract

**Background:**

Massive growth in human mobility has dramatically increased the risk and rate of pandemic spread. Macro-level descriptors of the topology of the World Airline Network (WAN) explains middle and late stage dynamics of pandemic spread mediated by this network, but necessarily regard early stage variation as stochastic. We propose that much of this early stage variation can be explained by appropriately characterizing the local network topology surrounding an outbreak’s debut location.

**Methods:**

Based on a model of the WAN derived from public data, we measure for each airport the expected force of infection (AEF) which a pandemic originating at that airport would generate, assuming an epidemic process which transmits from airport to airport via scheduled commercial flights. We observe, for a subset of world airports, the minimum transmission rate at which a disease becomes pandemically competent at each airport. We also observe, for a larger subset, the time until a pandemically competent outbreak achieves pandemic status given its debut location. Observations are generated using a highly sophisticated metapopulation reaction-diffusion simulator under a disease model known to well replicate the 2009 influenza pandemic. The robustness of the AEF measure to model misspecification is examined by degrading the underlying model WAN.

**Results:**

AEF powerfully explains pandemic risk, showing correlation of 0.90 to the transmission level needed to give a disease pandemic competence, and correlation of 0.85 to the delay until an outbreak becomes a pandemic. The AEF is robust to model misspecification. For 97 % of airports, removing 15 % of airports from the model changes their AEF metric by less than 1 %.

**Conclusions:**

Appropriately summarizing the size, shape, and diversity of an airport’s local neighborhood in the WAN accurately explains much of the macro-level stochasticity in pandemic outcomes.

**Electronic supplementary material:**

The online version of this article (doi:10.1186/s12879-016-1350-4) contains supplementary material, which is available to authorized users.

## Background

The world airline network (WAN) has massively increased the speed and scope of human mobility. This boon for humanity has also created an efficient global transport network for infectious disease [1, 2]. Pandemics can now occur more easily and more quickly than ever before. The accelerating emergence of novel pathogens exacerbates the situation [3]. Better understanding of global dispersal dynamics is a major challenge of our century [4]. Rapid assessment of an emerging outbreak’s dissemination potential is critical to response planning [5]. We do not know where the next pandemic threat might emerge. Mexico was not a prime candidate for an influenza outbreak, nor West Africa for Ebola. Preemptively mapping the pandemic influence of individual airports could contribute substantially to monitoring and response plans.

While exact relationships between the WAN and pandemic spread are difficult to model [2], simulation studies suggests that topological descriptors which describe epidemic outcomes on network models also have explanatory power for relationships between the topology of the WAN and pandemic spread [6, 7].

Observational studies of influenza [4, 8], malaria [9], and dengue fever [10] support this conclusion. Given the topology of a network, the minimal disease transmission rate which allows epidemics is given by the inverse of the spectral radius of a network’s adjacency matrix [11], and the typical outcome [12] and time course [13] of an epidemic follow a closed-form solution governed by the degree distribution of the network. The WAN’s topological structure is well characterized. It is a small-world, scale-free network with strong community structure, imposed partly by spatial constraints [14]. The majority of airports (70 %) serve as bridges which connect a densely interconnected core of 73 major transport hubs (2 %) to regional population centers and peripheral airports (28 %) [15]. Nodes which connect communities can be distinct from high-degree nodes within communities [16]. Since the WAN is designed to optimize passenger flow, the network’s temporal structure has little effect at time scales relevant for pandemic spread [17].

Topological descriptors of epidemic dynamics, however, can only describe typical outcomes. They do not describe the structure of the variation around the typical outcome, which is dismissed as stochastic when mentioned at all. Even within the constraints of a simple branching process model, empirical estimates of the probability of epidemic show substantial variation around the analytically derived solution. For example, the probability of a major outbreak in a discrete time Reed-Frost branching process with finite population is in theory the smallest solution to $x = e^{-R_{0}(1-x)}\phantom {\dot {i}\!}$, yet empirically observed probabilities from simulations of this same model can fall far from the theoretical value. Additional file 1: Figure S1 plots empirical vs theoretical values for this model.

Actual outcomes of emergent infectious diseases are crucially shaped by chance events in the early phases of their emergence [18]. Clear understanding of how seed location influences global outcomes would substantially improve public health planning [5].

The development of sophisticated, parameter-rich epidemic simulators provides powerful tools for exploring relationships between seed location and epidemic outcomes [19]. Common frameworks encompass demographic and mobility characteristics via either metapopulation [8, 20] or agent-based assumptions [21]. Careful tuning of these models has produced results which well match the spread of the 2009 influenza epidemic [19, 22]. Yet the complex interactions between model structure, input parameters, and estimation methods makes interpretation of model-based results challenging [18], especially when attempting to generalized to future outbreaks for which epidemic parameters are fundamentally unknowable. If, however, two radically different modeling approaches result in such high agreement both with each other and with reality [22] then the principal driver of outcomes should be expressible with a small parameter set [4]. Evidence suggests that simple probabilistic models incorporating local incidence, travel rates, and basic transmission parameters are sufficient to predict outcomes of complex metapopulation based simulations [23].

Recent theoretical work suggests that the apparent stochasticity in the early phases of a network-mediated epidemic process can be explained by the expectation of the force of infection of epidemic processes seeded from that node [24]. The aim of this study is to evaluate if this finding generalizes to realistic scenarios of WAN-mediated pandemic disease spread.

## Methods

### Defining and measuring airport expected force

Our model of the WAN is based on the 2014 release of the Open Flights database [25]. We selected all airports serviced by regularly scheduled commercial flights, resulting in a list of 3458 airports connected by 68,820 routes served by 171 different aircraft types. We simplify the network by replacing multiple routes between airports by a single edge whose weight is the sum of the available seats on all routes connecting the two airports, under the assumption that the aircraft type reflects the airline’s best judgment of the importance of the route. Aircraft seating capacity was estimated based on aircraft descriptions on worldtrading.net and airliners.net, using airlinecodes.co.uk to translate the International Air Transport Association (IATA) aircraft codes into aircraft type.

**Fig. 1:**
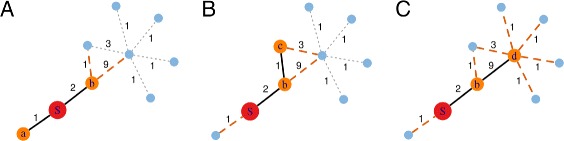
Computing AEF for a single airport. In the sample network show here, an epidemic can spread from the seed airport “S” (red) in the following ways: (*S*→*a*,*S*→*b*), (*S*→*b*,*S*→*a*) as shown in panel (**a**), both resulting in onward transmission strength of 1+9=10; (*S*→*b*,*S*→*c*) shown in panel (**b**), resulting in onward transmission strength of 1+3+9=13; (*S*→*b*,*S*→*d*) panel (**c**), resulting in onward transmission strength of (6∗1)+3=9. Transmission strength along a given edge is shown by the number beside the edge. The transmission *S*→*a* occurs with probability 1/3, after which *S*→*b* is the only remaining option. Transmission *S*→*b* occurs with probability 2/3; implying pattern (*S*→*b*,*S*→*a*) has overall probability 2/3∗1/11=2/33, (*S*→*b*,*S*→*c*) has overall probability 2/3∗1/11=2/33, (*S*→*b*,*S*→*d*) has overall probability 2/3∗9/11=18/33. The distribution of FoI values scaled by their probability is thus: 10∗1/3,10∗2/33,13∗2/33,9∗18/33, and the AEF of airport S is 1.09, the entropy of this series after normalization

The expected force of a network node is defined as the expectation of the force of infection (FoI) generated by an epidemic process seeded from the node into an otherwise fully susceptible network, after two transmission events and no recovery [24]. In a network model of disease spread, the FoI at any given time point is defined as the current number of edges between infected and susceptible nodes scaled by the base transmission rate of the disease; the standard generalization to weighted networks includes edge weights in the scaling. It is possible to enumerate all ways that two transmissions could occur from a single source node, and measure the FoI arising from each transmission pattern (up to the disease dependent scaling factor). The expected value of the FoI after two transmission events is the entropy of the distribution of possible FoI values. The definition extends to weighted networks, such as our model of the WAN, by including the influence of edge weights on the probability of observing a given pattern. Figure 1 illustrates the concept, which can be expressed mathematically as

$$AEF(i)=- \sum_{j=1}^{J} \bar{d_{j}} \log(\bar{d_{j}}) $$

where *A* *E* *F*(*i*) is Airport i’s Expected Force, *J* enumerates all possible ways to observe two transmissions seeded from *i*, *d*_*j*_ is the weighted degree of the *j* ^*th*^ transmission pattern multiplied by the probability that this pattern is observed given *J*, and $\bar {d_{j}}=d_{j} /\sum _{kj=1}^{J} d_{k}$ is the normalization of *d*_*j*_. We here further normalize AEF values to the range [0,100]. All computed AEF values are given in Additional file 2 and Additional file 1: Figure S2 shows their histogram.

### Simulation framework

Epidemic outcomes are generated using the GLEAMviz simulator [8, 20]. GLEAMviz integrates real-world global population and mobility data with an individual based stochastic mathematical model of the infection dynamics to produce realistic simulations of the global spread of infectious diseases. Spread within a local region follows user defined compartment models, while percolation between regions is modeled as a random processes based on real world airline and commuter data. Our basic experimental setup is to simulate the same disease model over a range of seed cities. The structure and parameters of the disease models are based on those which match the 2009 Influenza pandemic as reported in [8] and validated in [19], specifically, a Susceptible-Exposed-Infected-Recoverd (SEIR) model with transmission rate *β* specified below, latency rate *ε*=1/1.1 and recovery rate *μ*=1/2.5. Rates are expressed in units of days. The model further divides the infected compartment into three categories: asymptomatic, symptomatic travelers, and symptomatic non-travelers. When an individual moves from the exposed to infected compartment, they are placed in one of these three categories with equal likelihood. Non-symptomatic individuals have half the transmission rate of infected individuals. Symptomatic non-travelers contribute to local spread, but do not contribute to percolation between regions. Remaining parameters are left at their default values (occupancy rate: 90 %, time spend at destination: 8 h, commuting model: “data”, flight time aggregation: “month”).

The initial population distribution is 10 % of the seed city infected (symptomatic travelers) and the remainder of the (world) population susceptible. Seasonality effects are not included, since their influence varies both by time and geographic latitude, masking variability attributable to seed location.

GLEAMviz divides the world into sixteen regions. An outbreak is declared a pandemic on the day prevalence in at least three regions is greater than one per 100,000 inhabitants. The pattern of the results is invariant to thresholds in the range [0.1,100] per 100,000 inhabitants and to replacing the “three regions” criteria with “100 cities.” Results for each airport are reported in terms of the median over 20 runs (the maximum number supported by the public GLEAMviz client). If the threshold is not passed after 365 days (the maximum length supported by the public GLEAMviz client), we declare that no pandemic occurred.

### Defining and measuring epidemic stochasticity

For an outbreak to become a pandemic, its basic reproductive number *R*_0_ must surpass the basic epidemic threshold *R*_0_>1 needed to establish a disease in a local population by a sufficient amount to also overcome finite subpopulation size effects and diffusion rates to neighboring populations. A branching process approximation suggests that invasion thresholds in metapopulation models depend on the outbreak’s *R*_0_ value, the variance of the network’s degree distribution, and the mobility rate between subpopulations [7]. The GLEAMviz model specifies the last two values, reducing invasion thresholds to a function of *R*_0_. However, even a pure branching process shows substantial variability around the theoretical probability of achieving a large outbreak. For pandemics mediated by the WAN, the question of interest is how the invasion threshold varies for different seed airports. We empirically observe invasion thresholds on the WAN as follows. Ten seed airports are selected, one from each decile of the range of AEF values, see Table 1. Since our purpose here is to explore relationships between AEF and the minimal invasion threshold, we simplify the model in [8] by removing the subdivisions of the “infected” compartment. Including the asymptotic subcompartment would complicate the relationship between *β* and *R*_0_ [19], and including the non-traveler subcompartment would affect the mobility rate between subpopulations, which would impact *R*_0_ [19] and the invasion threshold [7]. Under this simplified model, the basic reproductive number is *R*_0_=*β*/*μ*, the transmission to recovery ratio. Keeping *μ* fixed, we vary *β* over the range [0.4, 0.5] and observe which seeds trigger a pandemic at each value under the simulation framework described above. The lower threshold *β*<0.4 corresponds to *R*_0_=1, the minimal level for a pandemic to occur. All simulations result in a pandemic for *β*≥0.475 (*R*_0_=1.19). Power analysis suggests that observations from 10 seed locations are sufficient to detect correlations between AEF and invasion thresholds of *ρ*=0.77 at a significance level of 0.05 with power of 0.80. Power calculations are based on the Z transformation of the correlation coefficient. They were made by specifying the number of samples and the indicated significance and power levels, assuming a two-sided test.

**Table 1:** Seed locations

Airport	City	Country	Pand.	*β*	AEF	t-core	Deg.	w. Deg.	Eigen.	w. Eigen.
SMK	St. Michael	USA	286	0.45	3	1	2	4	0.00	0.00
YFS	Fort Simpson	Canada	287	0.45	16	0	1	2	0.00	0.00
GTE	Groote Eylandt	Australia	300	0.45	27	3	3	4	0.00	0.00
SBH	Gustavia	France	346	0.42	31	7	6	9	0.01	0.00
PVH	Porto Velho	Brazil	295	0.45	40	4	8	19	0.00	0.00
BIS	Bismarck	USA	360	0.42	51	5	4	5	0.01	0.00
BES	Brest	France	284	0.42	62	24	9	15	0.05	0.00
XRY	Jerez	Spain	294	0.42	70	88	17	22	0.13	0.02
NRT	Tokyo	Japan	22	0.40	85	354	98	264	0.36	0.88
IST	Istanbul	Turkey	172	0.41	98	387	203	314	0.74	0.64

The following airports, shown by their IATA code, were selected as seed locations for testing relationships between AEF and invasion risk. The table additionally reports the number of days for an outbreak to reach pandemic status (“Pand.”) at the minimal observed transmission rate (*β*) for which a pandemic occured, along with each airport’s AEF, t-core, (un)weighted degree, and (un)weighted eigenvalue centralities

Often, diseases of concern are known to be competent of invading the network. Here, the outcome of interest is not if a pandemic occurs, but rather how long until an outbreak reaches pandemic status. We measure relationships between AEF and time to pandemic status as follows. One hundred world airports were chosen such that they evenly cover the range of measured AEF values.

To better replicate a real pandemic, we use the three category infected compartment model as per [8], also setting the base transmission rate to *β*=0.8383 as in that publication. Translating this rate into an *R*_0_ value requires accounting for the reduced transmissibility of the asymptotic compartment, *R*_0_=*β*/*μ*[*r*_*β*_ *ρ*_*a*_+(1−*ρ*_*a*_)], where *r*_*β*_ is the reduction in infectivity for the asymptomatic compartment and *ρ*_*a*_ is the probability that an infectious person is asymptomatic [19]. This implies our simulations are based on *R*_0_=1.75, well above the minimal invasion threshold of *R*_0_≈1.19 determined empirically above.

For each seed location, we observe both the number of days until pandemic status is reached and the number of days until peak global incidence. Both outcomes are highly correlated, since once pandemic status is achieved further disease development is determined by network topology. The purpose of measuring peak global incidence is that this measure is unambiguous, while any definition of “first day of pandemic status” is somewhat arbitrary. A Shapiro-Wilks test of the observed times to peak global incidence suggests that this data is approximately normally distributed (*p*=0.69 under the null hypothesis that the data is normally distributed), while the distribution of observations of first day of pandemic status is right-skewed (*p*=0.04).

Relationships between outcomes and AEF are measured by Pearson correlation. We additionally test correlations to weighted and unweighted versions of each airport’s betweenness, degree, and eigenvalue centralities, and also to Verma et al’s t-core, a variant of the k-core which counts triangles [15].

These well-known centrality indices can be briefly defined as follows. Betweenness considers all shortest paths which connect all possible pairs of nodes in the network, and counts how many of these pass through the node of interest. Degree counts the number of edges connected to a node. Eigenvalue centrality counts the number of infinitely long paths originating from a given node. Core-based algorithms recursively strip off nodes from the periphery of the network based on some criteria which is re-evaluated on each round; a node’s core centrality indicates the round at which is removed. The t-core uses the count of how many network triangles a node contributes to as its removal criteria; a node which does not participate in any triangles would be removed on the first round, then all nodes which participate in only one triangles, etc. For completeness, Additional file 1: Figures S3–S6 show plots of AEF against betweenness, degree, eigenvalue, and t-core.

As noted above, outcomes are based on the median daily prevalence over 20 runs. The public GLEAMviz client also indicates the 95 % confidence interval of daily prevalence. This allows us to estimate confidence bounds on the the time until an outbreak achieves pandemic status, since our definition of pandemic status is derived from prevalence levels. Analysis of these interval provides further insight into the robustness of the correlation results. Further, the size of the interval can be considered as an additional form of epidemic stochasticity. Accordingly, we also compute relationship between AEF and this observation. Since the date of peak global incidence is somewhat independent of the magnitude of the peak, the GLEAMviz output does not easily lead to a meaningful way to determine confidence bounds for this outcome.

### Robustness of AEF to sampling error

The robustness of AEF values is examined by observing their relative change while progressively degrading the model WAN from which they are derived. The network is degraded by removing from one to 15 percent of U.S. airports from the network along with their associated edges. The AEF of all remaining world airports is computed. Community-based analysis of the WAN suggest that US airports form one large community [15, 16]. The AEF is derived from the local neighborhood of the airport. Restricting degradation to a single network community lets us evaluate both regional and global effects of degradation on the AEF. Three different random removal schemes were used: uniform over all airports, selection weighted by airport degree (here defined in terms of the seating capacity on all outbound routes from that airport), selection weighted by AEF. The resulting AEF values are compared with the original AEF values. We record the number of airports whose degraded AEF departs from its original AEF by more than 1 % and by more than 5 %. Reported results are the averaged over ten runs, and show the amount of degradation for both U.S. and non-U.S. airports.

### Ethics statement

None of the research reported in this paper involved human or animal subjects, or human or animal data.

## Results

**Fig. 2:**
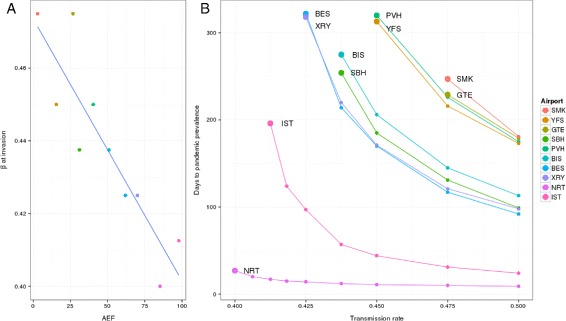
AEF and invasion threshold. Higher AEF is associated with a lower transmission rate needed to trigger pandemics (panel **a**), as well as shorter delay until the outbreak reaches pandemic status (panel **b**). In panel (**b**), the large dots mark the lowest transmission rate for which a pandemic occurred, for each city, and correspond to the points used to generate the regression line in panel (**a**). Cities are listed in order of increasing AEF. See also Table 1

The AEF of the seed location is strongly predictive of an outbreak’s invasive threshold as shown in Fig. 2 and Table 1. The correlation between AEF and the minimal observed transmission rate at which it first became pandemically competent was 0.90 (95 % confidence interval: 0.98,0.62). Tokyo was a notable outlier, achieving pandemic competence earlier than predicted from its AEF value.

**Fig. 3:**
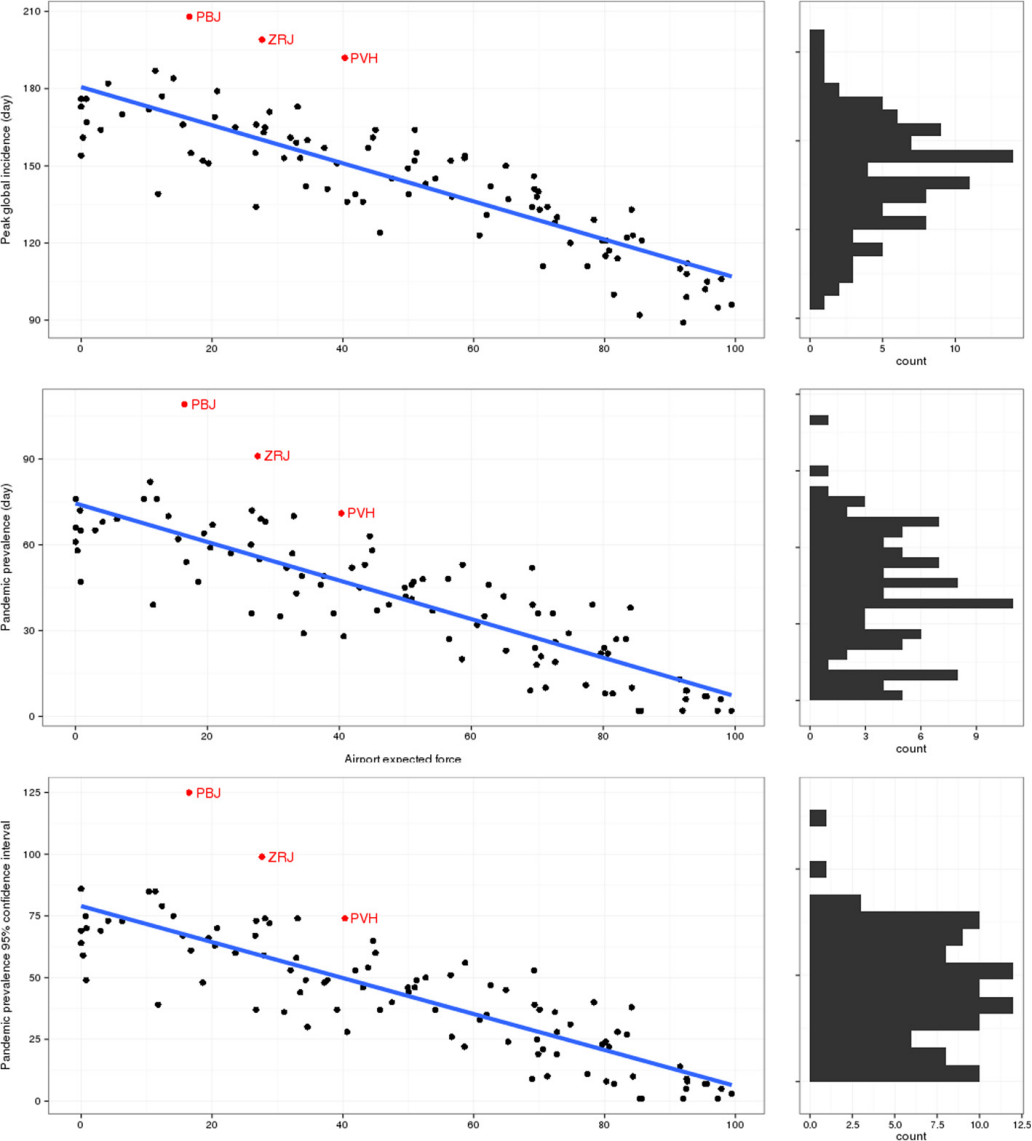
AEF and time to pandemic. Airport expected force has strong correlation to the rate and to variation in the rate at which a simulated epidemic becomes pandemic, outcomes which otherwise appears stochastic, as shown in the histograms in the right column. The top panel shows days to peak global incidence. The middle panel shows days until the disease achieves pandemic prevelance, and the lower shows the range of the confidence interval for this measure. Three outlers are clearly visible in the top panel. These are are PBJ (Paama Island, Vanuatu), ZRJ (Round Lake, Canada), and PVH (Porto Velho, Brazil), and are colored red in all three plots

**Fig. 4:**
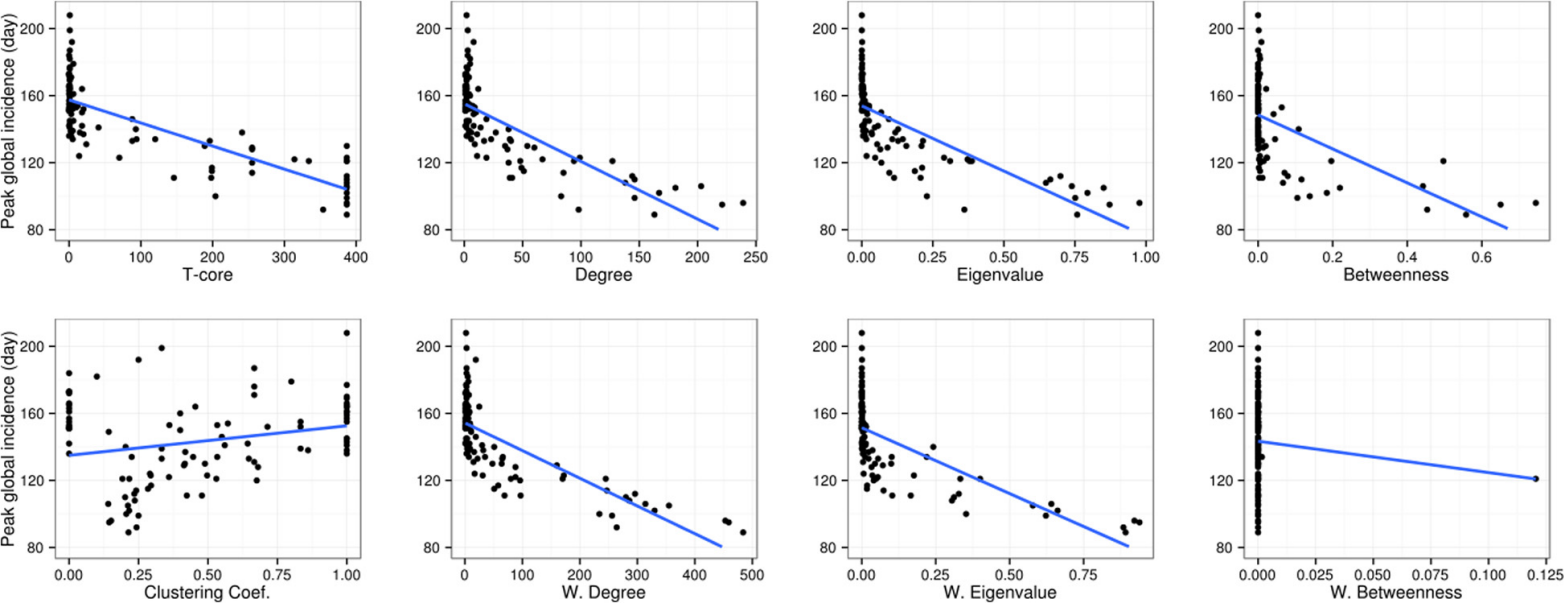
Centrality measures and time to peak global incidence Common network centrality measures do not well explain variation in the timing of peak global incidence. In each scatterplot, the y axis indicates the day of peak global incidence for an infection started from each airport, and the x axis indicates the centrality value of the starting airport. The blue line inidcates the best linear fit to the data. The centrality measure is shown under each plot. The abbreviation “W” indicates the weighted version of the measure

AEF was also strongly correlated with the delay until an outbreak became a pandemic. Correlation was 0.84±0.058 to the day pandemic status was achieved, and 0.85±0.056 to the day of peak global incidence, see Fig. 3. AEF is significantly and more strongly correlated to either epidemic outcome than any of the comparison network centrality measures, see Table 2 and Fig. 4.

**Table 2:** Correlation and 95 % confidence interval between a suite of network centrality measures (rows) and days until both pandemic status and peak global incidence

Measure	Pandemic	c.i.	Peak	c.i.	
AEF	–0.84	±0.06	–0.85	±0.06	
t-core	–0.77	±0.09	–0.79	±0.08	
Degree	–0.72	±0.10	–0.75	±0.09	
W degree	–0.69	±0.11	–0.75	±0.09	
Eigenvalue	–0.70	±0.11	–0.74	±0.09	
W eigenvalue	–0.66	±0.12	–0.69	±0.11	
Betweenness	–0.54	±0.14	–0.56	±0.14	
W betweenness	–0.17	±0.20	–0.09	±0.20	
Clustering coef.	0.28	±0.19	0.24	±0.19	
W Clust. coef.	0.27	±0.19	0.24	±0.19	

The abbreviation “W” indicates the weighted form of the measure

The confidence values surrounding the median time to pandemic showed an interesting pattern. In every case the value at the low end of the interval was equivalent to the median value. This indicates that most runs showed no variation, and that variation, when it occurred, was always in the form of slower spread. Correlation between AEF and the size of the interval was 0.84±0.060. This indicates that AEF explains not only the power with which an airport can seed a pandemic, but also the variation in that power over multiple seeding events. Typical sizes of the confidence intervals ranged from one to three days for airports with high AEF to circa 80 days for airports with low AEF, see Fig. 3.

**Fig. 5:**
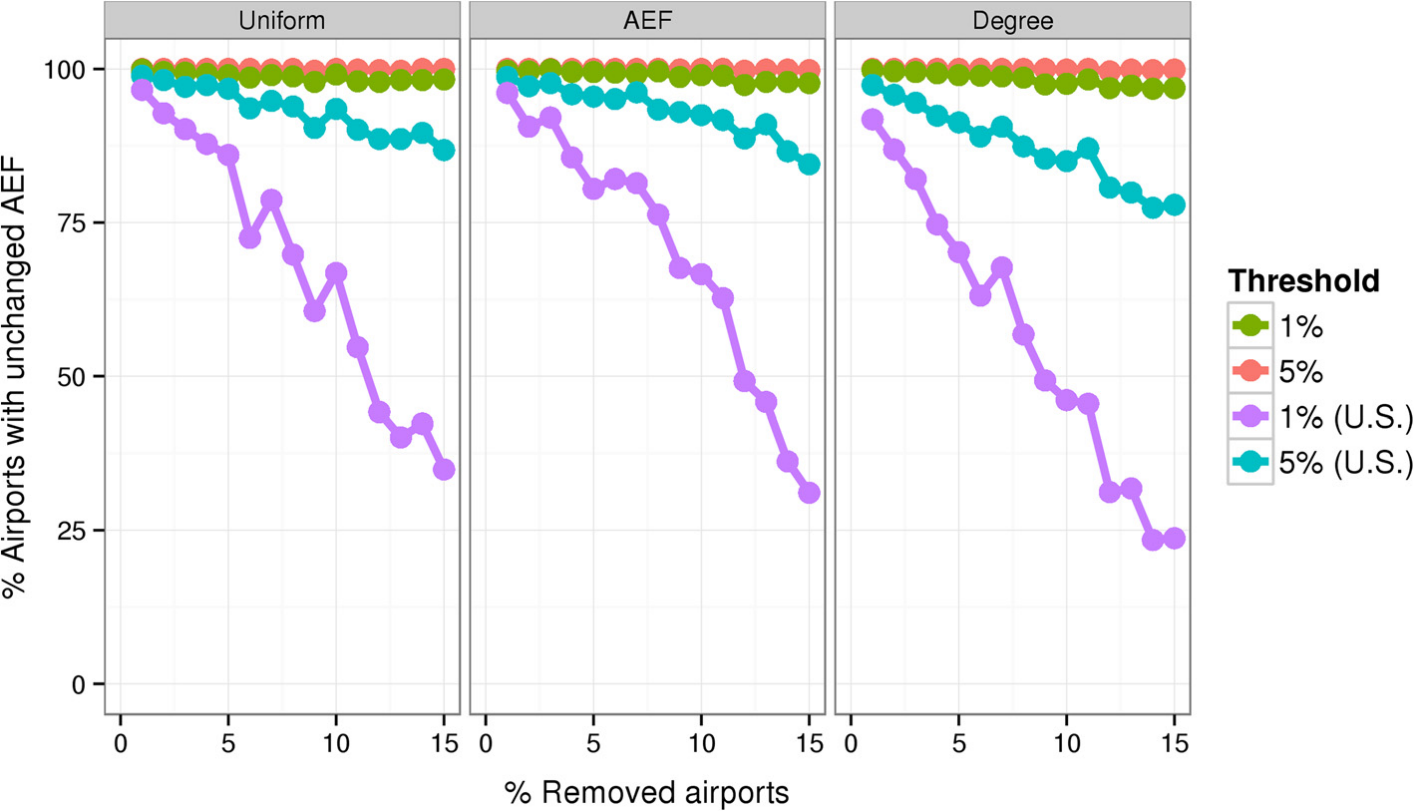
Robustness of AEF values to model degradation. For non-US airports, almost all AEF values are unaffected (defined as less than 1 %/5 % change) as US airports are removed from the model. While many US airports are affected at the 1 % level, few show more than 5 % change in AEF. The network is degraded by removing airports with selection probabilities weighted uniformly, by AEF, and by degree

The AEF proved robust to incomplete sampling. Degradation was most severe when airports were preferentially removed based on degree. Still, only three percent of non-U.S. airports showed more than 1 % change in their computed ExF values when applying this scheme at the highest noise level. Even within the United States, only 22 % of AEF values changed by more than 5 %. See Fig. 5.

## Discussion

In all cases, AEF explains much of the variation in epidemic outcomes, suggesting that the early development of a pandemic is not stochastic, but rather strongly structured by the local connectivity of the seed location. The ability of the AEF to summarize this connectivity contributes substantially to our understanding of the role of individual airports in pandemic diffusion. These results are in harmony with other recent work claiming that relative arrival times of WAN-mediated pandemics are independent of disease-specific parameters [4] and that a simple branching process model is as capable of describing early developments as complex metapopulation simulations [23].

Degradation of the network had, in general, limited effect on airport AEF values. Wrong information regarding a specific node could, however, produce a misleading AEF value for that airport. Epidemics seeded from airport PBJ (Paama Island, Vanuatu) took longer than expected to achieve pandemic status. This airport is probably mischaracterized in the Open Flights database, as flights to this simple grass strip are not shown on the Vanuatu airlines online booking system (http://www.airvanuatu.com/, last visited 23 March 2015). In the opposite direction, Narita Airport (NRT, Tokyo, Japan) showed significantly greater pandemic risk than predicted by its AEF. This could be due to Japan’s intense population density combined with high local mobility, factors captured in the GLEAMviz simulator but not the Open Flights database.

Two outliers highlight a structural blind spot of the AEF metric. Epidemics seeded from airports ZRJ (Round Lake, Canada) and PVH (Porto Velho, Brazil) took longer than expected to achieve pandemic status. ZRJ is part of a small but locally dense community of airports serving first nation communities in Canada. This community has limited connectivity to the rest of the WAN, and ZRJ is three flights distant from any airport outside this community (Winnipeg’s James Armstrong Richardson Airport YWG, Chicago Midway MDW, Toronto Pearson YYZ). Likewise, PVH is two flights from any of Brazil’s international transport hubs. The AEF is here derived from an airport’s two-hop neighborhood, meaning for certain airports it is unaware of these network community boundaries. This limitation could perhaps be overcome by instead computing AEF based on a three-hop neighborhood. Given, however, that the WAN’s effective diameter is four hops, and the general good performance of the AEF, it is not clear that such an extension would substantially improve results globally.

Airport expected force summarizes the size, density, and diversity of each airport’s neighborhood in the WAN. The innovation of the AEF is in defining airport influence from epidemiological first principles rather than from network theoretical definitions of importance. The significance of this is profound. Network theoretic measures encode one particular assumption about how topology reflects influence. They are only valid for networks, or network regions, where that assumption holds [26]. In contrast, measuring influence as the expected force of infection gives a measure whose theoretical validity is independent of specific network topology [24].

Airport degree is not a good descriptor of pandemic outcomes. Guimera *et al* noted that high degree does not well correlate to high centrality in the WAN [16], because it does not incorporate neighborhood structure. Nor does low degree correlate to an airport’s connection to the wider network, as illustrated by comparing Sweden’s Linköping City Airport (LPI) to Alaska’s Huslia Airport (HSL). HSL has four outbound routes which connect to other rural Alaskan airports. LPI has only one outbound route, which connects to Amsterdam Schipol.

The classical way to account for a neighbor’s onward connectivity is to cast centrality as an eigenvalue problem. The validity of this approach has recently come into question, with luminaries such as Newman showing that eigenvalue-based centralities tend to concentrate most of the centrality score on only a few nodes [27], and Pastor-Satorras and Castellano showing that replacing the graph adjacency matrix with a non-backtracking variant, the solution proposed in [27], does not resolve the problem [28]. While we observe these effects in our model WAN, eigenvalue centrality still provides good fit to epidemic outcomes for those airports with centrality high enough to distinguish them from the large mass of low-centrality airports (see Table 2). Similar findings have been previously reported, with one study showing that adding the weighted mean geographic distance between the source airport and its immediate neighbors to a weighted eigenvalue centrality yeilds a metric in qualitative agreement with the variance in spatial position of infected agents measured on day ten of simulations seeded from 40 major US airports [29].

Verma *et al* propose characterizing airports based on the number of network triangles they take part in, the t-core [15]. The t-core is not presented as a method to quantify epidemic spread. It is rather a variant of the k-shell algorithm, which is designed to precipitate away outer layers of a network in order to identify core network groups [30]. We find that the t-core has the second highest correlations to epidemic outcomes after AEF. Plotting airport t-core against epidemic outcomes shows that this is a result of its ability to successfully segment the WAN into core and periphery, see Fig. 4. Thus t-core and AEF capture complementary aspects of an airport’s role in the WAN.

The model of the WAN used to compute AEF differs from the GLEAMviz simulator mobility model. The WAN model replicates the airline network only, and thus regards each airport as a separate entity. GLEAMviz is designed instead to model human mobility patterns between regions. Accordingly, GLEAMviz regards large metropolitan centers such as London or New York City as a single transport hub regardless of the number of airports which serve that region, and also includes commuter traffic over road networks. These differences impact our analysis; we test the correlation between the AEF value for i.e. London Heathrow airport to disease spread simulated from the entire London region, which includes four airports. This could explain why simulated spread from high AEF airports, which tend to be associated with major metropolitan centers, is uniformly faster than the value implied by the linear correlation between AEF and time to pandemic (see Fig. 3). For example, London Heathrow has an AEF of 92, compared to Paris’s Charle de Gaul’s 97. Simulated pandemics seeded from London achieve maximum global incidence six days earlier than those seeded from Paris. The two models also differ in how they weight different flight routes, and perhaps even in which routes are included. The general high accuracy of the AEF in predicting GLEAMviz simulation results, despite these differences, suggests that the AEF will generalize well to the real world, which also departs in important ways from any existing model. This suggestion is re-enforced by the results of the robustness analysis, which show that clear omissions in the underlying model have only minimal effect on estimated AEF values.

The applicability of the AEF could be extended by modifying it to allow for varying transmissibility at individual airports. Such an extension would allow it to express differences in i.e. competent vector species populations or health care system readiness at different world locations. Since the AEF is the expectation of the force of infection, such an extension merely requires modifying the calculation of each transmission pattern’s force of infection along with the probability of that specific pattern occurring. Both criteria can be met by adjusting edge weights in the underlying network model, implying that this extension could be implemented using the same framework as outlined in the current work. It would also be interesting to apply the expected force framework to disease spread through the world shipping network, a major transport system for several vector born pathogens along with their vector species [1]. The approach could also be tested on more local transmission network models, such as contacts in a hospital ward [31] or city-wide mobility data acquired from i.e. mobile phones [32, 33].

## Conclusion

An outbreak’s debut location is highly influential in its ability to become a pandemic threat. The AEF metric succinctly captures this influence, and can help inform monitoring and response strategies.

## Additional files

Additional file 1 **Supplementary figures.** This supplement presents figures which further explore topics raised in the main text. (PDF 878 kb)

Additional file 2 **Airport AEF values.** This CSV file gives the AEF of the airports as calculated and used in the current study. Airports are indexed by IATA code, and also by city and country. AEF values are normalized to the range 0,100. (CSV 131 kb)

## Abbreviations

AEF: airport expected force
FoI: force of infection
IATA: international air transport association
SEIR: susceptible exposed infected recovered
WAN: world airline network
