# Supplementary material for: Measuring the potential of individual airports for pandemic spread over the world airline network
Source: BMC Infect Dis. 2016 Feb 9;16:70. doi: 10.1186/s12879-016-1350-4 (PMC4746766; doi:10.1186/s12879-016-1350-4)
Supplement: Supplementary file 1 — Supplementary figures. This supplement presents figures which further explore topics raised in the main text. (PDF 878 kb) [file 12879_2016_1350_MOESM1_ESM.pdf]

Measuring the potential of individual airports for pandemic spread over the World Airline Network

Supplemental figures

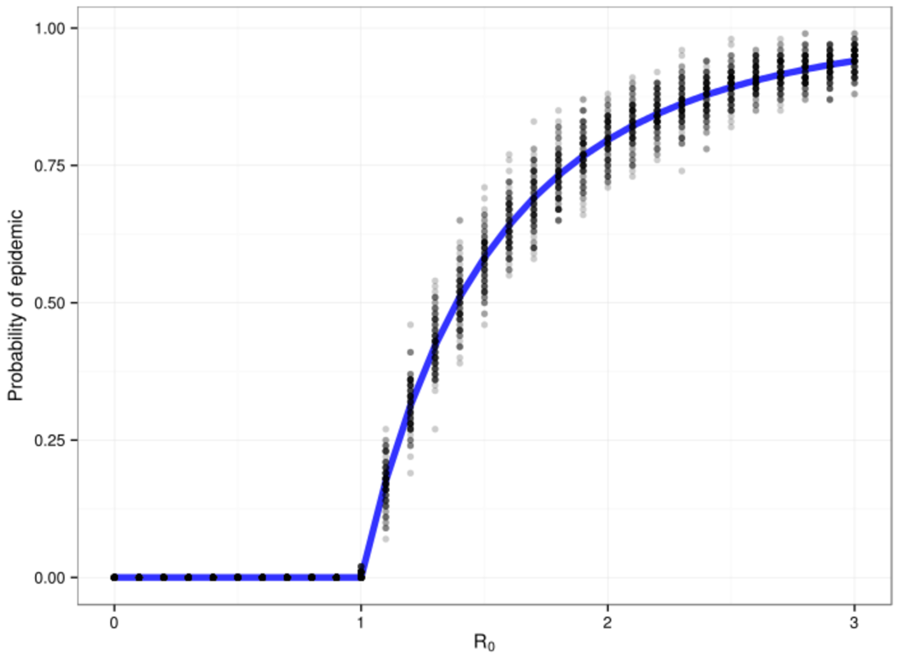

Figure S-1: **Stochastic variation around the probability of a major outbreak under a simple branching process model.** In a discrete time Reed-Frost branching process with finite population, the probability of a major outbreak is the smallest solution to $x = e^{-R_0(1-x)}$, shown above as the solid blue line, where R_0 is the base reproductive number of the disease process. The black dots show empirically observed probabilities from simulations of the same model. Each dot is the observed fraction of major outbreaks out of 100 simulated outbreaks for a given value of R_0. For each value of R_0, 100 dots are generated.

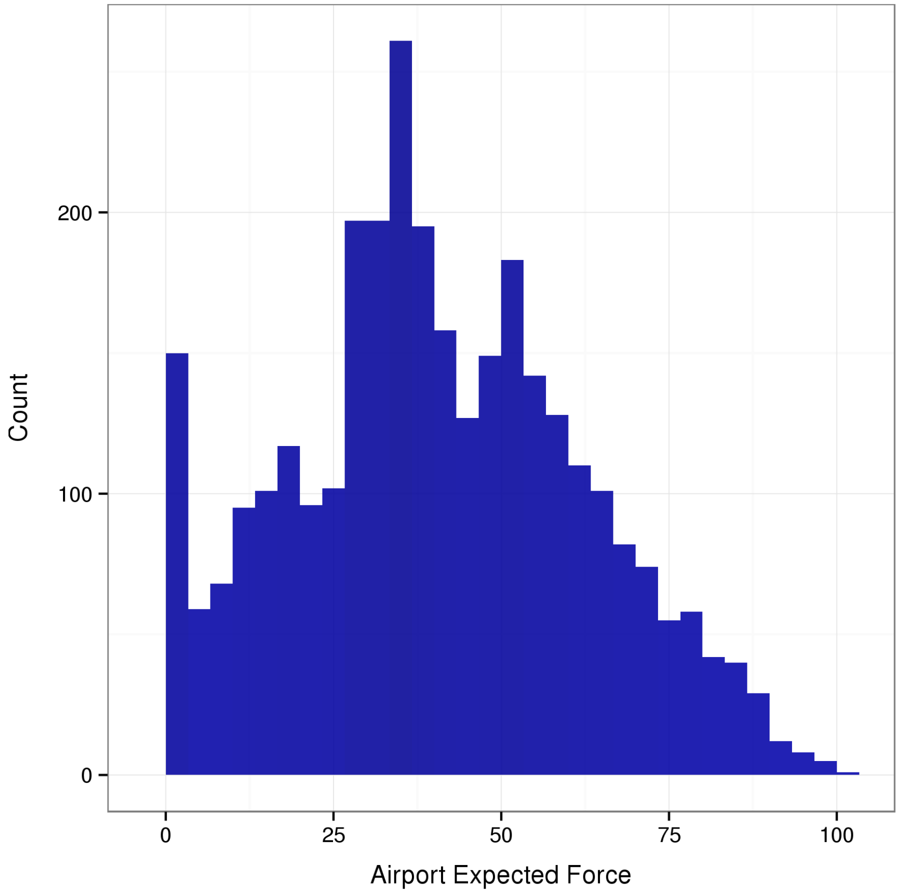

Figure S-2: **Histogram of AEF values.** Airports at the extreme periphery of the WAN will have AEF of zero; our model of the WAN has 89 such airports. All AEF values are given in Additional File 2.

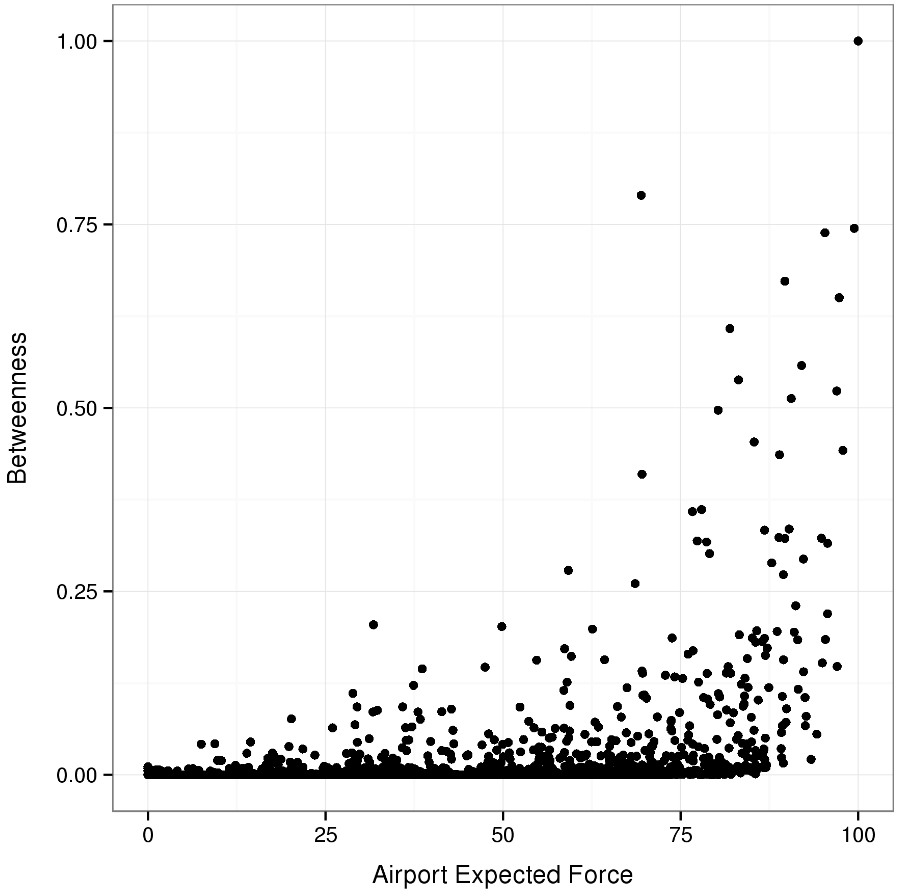

Figure S-3: **AEF vs Betweenness centrality.** AEF shows little relation to betweenness centrality.

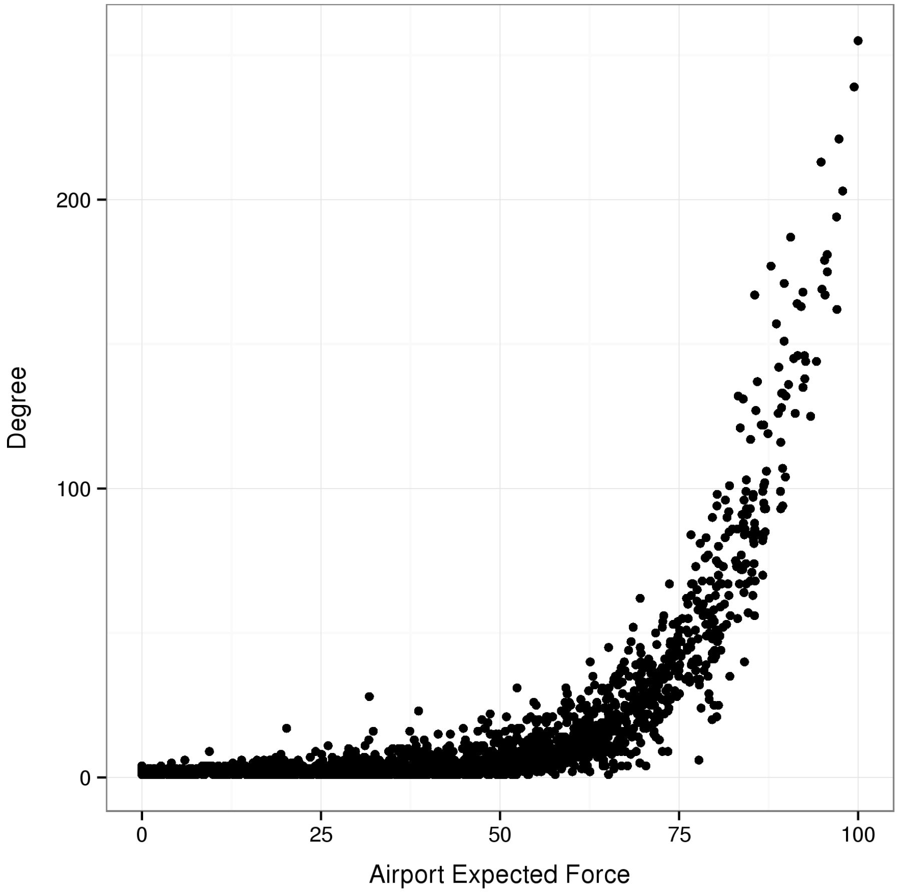

Figure S-4: **AEF vs Degree centrality.** The relationship between AEF and degree follows from the definition of AEF. If an airport has degree=n_1 first-degree neighbors and n_2 second-degree neighbors, then the number of terms in the summation which determines AEF scales at $\approx n_1^2+n_2$. Comparison here is to unweighted degree (i.e. number of outbound flight routes from a given airport, with no regard for the number of flights/seating capacity on each route).

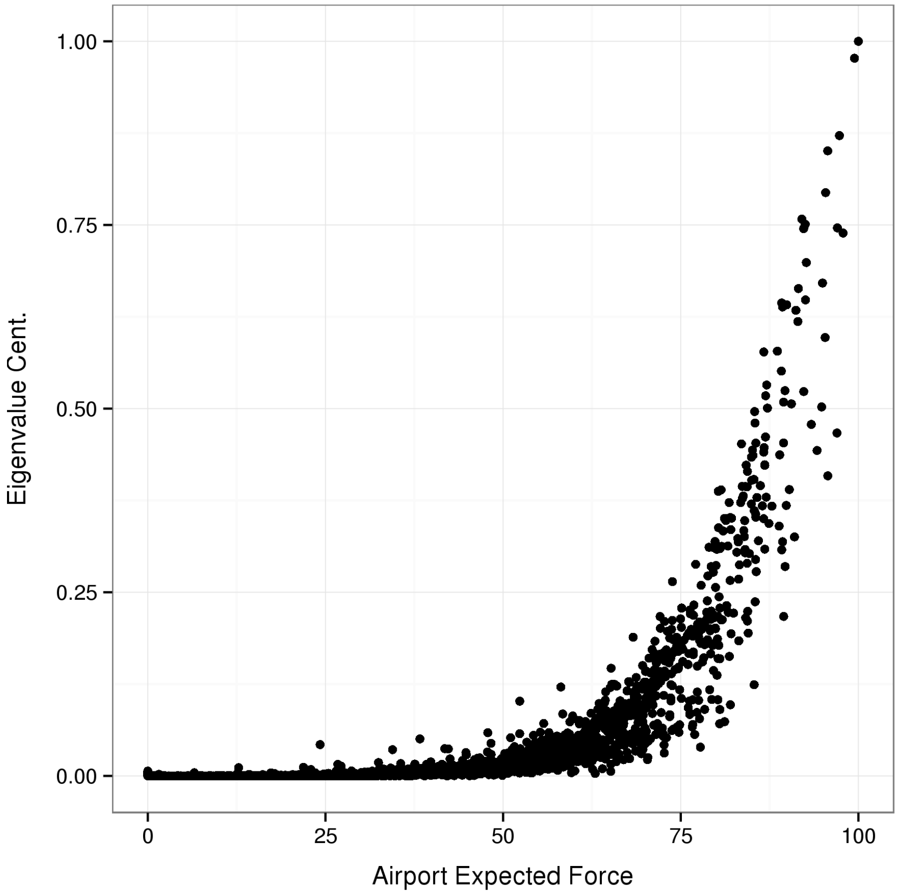

Figure S-5: **AEF vs eigenvalue centrality.** The dense structure of the airline network implies that AEF and eigenvalue centrality show strong rank order correlation. AEF, however, does not suffer from the localization effects observed in the eigenvalue centrality (many values near zero). Comparison here is to unweighted eigenvalue centrality (i.e. based on the number of outbound flight routes from a given airport, without weighting for the number of flights/seating capacity on each route).

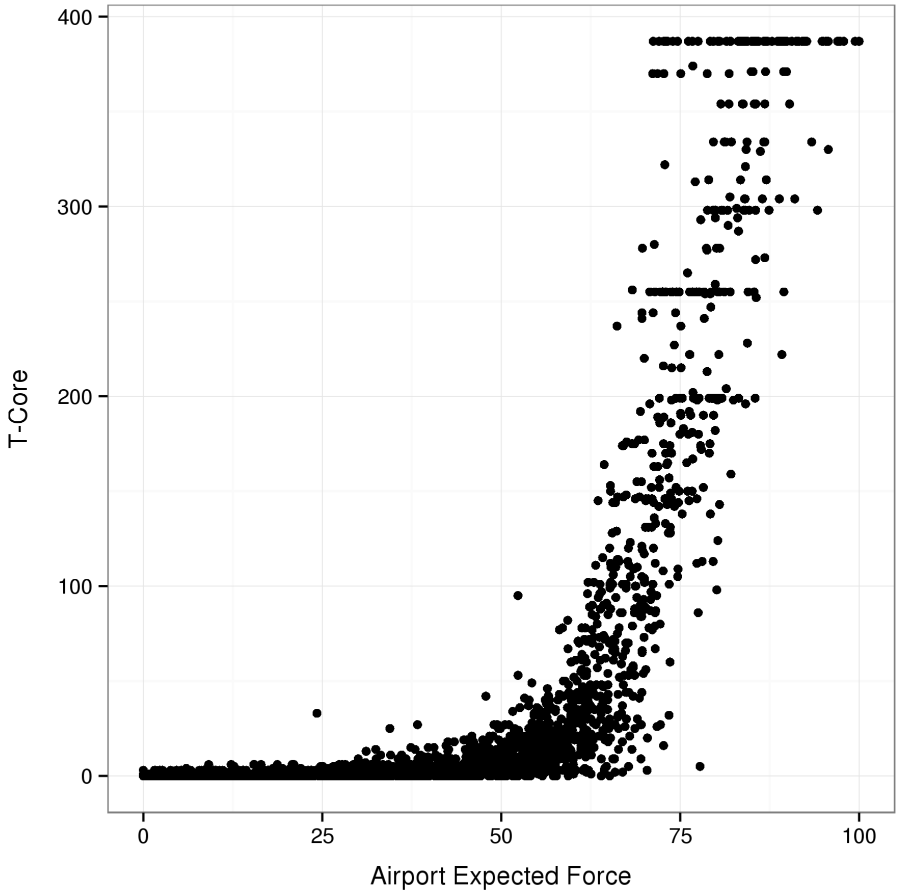

Figure S-6: **AEF vs T-core.** The t-core is designed to identify the coreelements of a network, rather than to measure node influence. In the WAN, however, which has a single core, an airports core-ness well corresponds to its epidemic potential.
